# Supplementary material for: Chromothripsis during telomere crisis is independent of NHEJ, and consistent with a replicative origin
Source: Genome Res. 2019 May;29(5):737–49. doi: 10.1101/gr.240705.118 (PMC6499312; doi:10.1101/gr.240705.118)
Supplement: Supplemental Material [file supp_gr.240705.118_Supplemental_file_1.zip › contigs/annotated_contigs/DB77/contig.2.DB77_length_540_mean_cov_8.0537037037.docx]

**DB77_length_540_mean_cov_8.0537037037**

```
TGTGAATGAATCCAATCTCTAATTCTAATTTTTTTATTATCTTTTTTATTTTTTATTTATTTAGTGTTTTTGAGATGGAGTTTCGCTCT
 >chr9:30560441-30560664 + E=1e-116
GTCACCCAGGCTGGAGCACAGTGGCGTGAACTCCGCTCACTGCAACCTCTGCCTCCCGGGTTCGCGCCATTCTCCCGCTGCAGCCTCCC

GAGTAGCTGGGACTACAGGCGCCCGCCATCATGCCTGGCTAA|GTT|GGGGAAATTACAACTGGGTAAGTTGTTGAAACGGTGTGCTTT
                                           >chr9:30561266-30561587 + E=9e-177
ATCTGGACATCCATTTATTGCTTCACCTGTATAGACCCTAACTCTAACAGGGAACACTTTAGATCTCTTTTTATTAATGTCAACTCTGA

CCCTTTGTTTGATAGTCCTTGAAATGTCTTAGTATTTTTCTTTCCCCAGAAAAATTCAACTGAATAAATGGCTATACGTCTCTTTGGGG

CAAAAGTGGAGTAATCATTACAAGATATAATTGCTTTAATTTTGTACAGCTATTTGATTTAAAAACTGGCCACGTAATCAACTAATTTC

ATATCTGA
```
